# Supplementary material for: The Feasibility and Preliminary Efficacy of an eHealth Lifestyle Program in Women with Recent Gestational Diabetes Mellitus: A Pilot Study
Source: Int J Environ Res Public Health. 2020 Sep 28;17(19):7115. doi: 10.3390/ijerph17197115 (PMC7579575; doi:10.3390/ijerph17197115)
Supplement: Supplementary file 1 [file ijerph-17-07115-s001.pdf]

Supplementary Table 1. Frequency of responses to process evaluation questions for the intervention groups (high personalisation, *n* = in a 6-month pilot randomised controlled trial for women with recent GDM who were at risk of developing T2D.

**Program element**	**Question**	**3 months**[a] *n* = 25	**6 months** *n* = 21
		% (*n*)	% (*n*)
**Australian Eating Survey report**	I read/saw the Australian Eating Survey report		
	Yes	88 (22)	81 (17)
	No	12 (3)	19 (4)
	Easy to understand		
	Agree/Strongly agree	89 (17)	88 (15)
	Neutral	0	6 (1)
	Disagree/ Strongly disagree	11 (2)	6 (1)
	Helped identify areas in my diet to improve		
	Agree/Strongly agree	95 (18)	100 (17)
	Neutral	5 (1)	0
	Helped identify areas in my diet where I am doing well		
	Agree/Strongly agree	100 (19)	100 (17)
	Helped me set diet goals		
	Agree/Strongly agree	79 (15)	82 (14)
	Neutral	21 (4)	12 (2)
	Disagree	0	6 (1)
**Physical Activity report**	I read/saw the Physical Activity Report		
	Yes	82 (18)	81 (17)
	No	18 (4)	19 (4)
	Easy to understand		
	Agree/Strongly agree	94 (17)	94 (16)
	Neutral	6 (1)	6 (1)
	Helped me identify areas in my physical activity to improve		
	Agree/Strongly agree	89 (16)	88 (15)
	Neutral	11 (2)	12 (2)
	Helped me identify areas in my physical activity where I am doing well		
	Agree/Strongly agree	78 (14)	88 (15)
	Neutral	22 (4)	12 (2)
	Helped me set physical activity goals		
	Agree/Strongly agree	78 (14)	76 (13)
	Neutral	22 (4)	18 (3)
	Disagree	0	6 (1)
**Body Balance Beyond website: Content**	I accessed the BBB website		
	Yes	91 (20)	57 (12)
	No	9 (2)	43 (9)
	Provided me with useful information about my diabetes risk		
	Agree/Strongly agree	95 (19)	92 (11)
	Neutral	5 (1)	8 (1)

Provided me with useful information about healthy eating		
Agree/Strongly agree	90 (18)	75 (9)
Neutral	10 (2)	25 (3)
Provided me with useful information about physical activity		
Agree/Strongly agree	85 (17)	75 (9)
Neutral	15 (3)	25 (3)
Provided me with useful information about weight-loss strategies		
Agree/Strongly agree	65 (13)	67 (8)
Neutral	35 (7)	33 (4)
Provided me with useful information about other wellbeing strategies		
Agree/Strongly agree	55 (11)	58 (7)
Neutral	45 (9)	42 (5)
Helped me to attain my goals		
Agree/Strongly agree	35 (7)	25 (3)
Neutral	55 (11)	17 (2)
Disagree	10 (2)	58 (7)
BBB website was motivating		
Agree/Strongly agree	30 (6)	25 (3)
Neutral	45 (9)	17 (2)
Disagree	25 (5)	58 (7)
Made me feel accountable		
Agree/Strongly agree	35 (7)	50 (6)
Neutral	30 (6)	17 (2)
Disagree	35 (7)	33 (4)
Easy to navigate		
Agree/Strongly agree	90 (18)	75 (9)
Neutral	5 (1)	17 (2)
Disagree	5 (1)	8 (1)
Visually appealing		
Agree/Strongly agree	80 (16)	92 (11)
Neutral	20 (4)	8 (1)
Satisfaction with managing my risk		
Satisfied/Very satisfied	60 (12)	58 (7)
Neutral	35 (7)	33 (4)
Unsatisfied	0	8 (1)
Satisfaction with developing my plan		
Satisfied/Very satisfied	65 (13)	58 (7)
Neutral	30 (6)	25 (3)
Unsatisfied	5 (1)	8 (1)
Satisfaction with healthy eating resources		
Satisfied/Very satisfied	75 (15)	67 (8)
Neutral	25 (5)	33 (4)
Satisfaction with physical activity resources		
Satisfied/Very satisfied	70 (14)	67 (8)
Neutral	30 (6)	33 (4)
Satisfaction with wellbeing resources		
Satisfied/Very satisfied	70 (14)	58 (7)
Neutral	20 (4)	42 (5)
Frequency using the Goal Setting Module		

	At least once per week	15 (3)	8 (1)
	At least once per month	70 (14)	42 (5)
	Never	15 (3)	50 (6)
**Body Balance Beyond website: Goal Setting Module**	Made it easy to set weight-related goals		
	Agree/trongly agree	82 (14)	100 (6)
	Neutral	18 (3)	0
	Made it easy to set my nutrition goals		
	Agree/Strongly agree	88 (15)	100 (6)
	Neutral	12 (2)	0
	Made it easy to set my exercise goals		
	Agree/Strongly agree	94 (14)	100 (6)
	Neutral	6 (1)	0
	I liked being able to select from pre-written goals		
	Agree/Strongly agree	82 (14)	100 (6)
	Neutral	18 (3)	0
	Email summary of my goals was motivating		
	Agree/Strongly agree	59 (10)	83 (5)
	Neutral	24 (4)	0
	Disagree	18 (3)	17 (1)
	Email summary of my goals was useful		
	Agree/Strongly agree	77 (13)	100 (6)
	Neutral	18 (3)	0
	Disagree	6 (1)	0
**Video coaching sessions: Dietitian**†	Provided me with useful information about nutrition		
	Agree/Strongly agree	100 (13)	-
	Increased my confidence to improve my diet		
	Agree/Strongly agree	85 (11)	-
	Neutral	15 (2)	
	Helped me to achieve my goals		
	Agree/Strongly agree	85 (11)	-
	Neutral	15 (2)	-
	Made me feel accountable		
	Agree/Strongly agree	100 (13)	-
	Strategies addressed barriers to healthy eating		
	Agree/Strongly agree	77 (10)	-
	Neutral	15 (2)	-
	Disagree	8 (1)	-
	Email summary of goals and strategies was useful		
	Agree/Strongly agree	100 (13)	-
	Video coaching sessions were useful		
	Agree/Strongly agree	92 (12)	-
	Neutral	8 (1)	-
	Video coaching was easier than attending in person		
	Agree/Strongly agree	92 (12)	-
	Disagree	8 (1)	-
	Number of sessions		
	About right	46 (6)	-
	Would have preferred more contact	54 (7)	-
	Duration of sessions		
	Too short	15 (2)	-
	About right	85 (11)	-

Video coaching sessions: Exercise physiologist[b]	Provided me with useful information about exercise		
	Agree/Strongly agree	100 (13)	-
	Increased my confidence to improve my activity behaviours		
	Agree/Strongly agree	92 (12)	-
	Neutral	8 (1)	-
	Helped me to achieve my goals		
	Agree/Strongly agree	85 (11)	-
	Neutral	15 (2)	-
	Motivated me to be more active		
	Agree/Strongly agree	100 (13)	-
	Made me feel accountable		
	Agree/Strongly agree	92 (12)	-
	Disagree	8 (1)	-
	Addressed barriers preventing me from being active		
	Agree/Strongly agree	77 (10)	-
	Neutral	23 (3)	-
	Video coaching sessions were useful		
	Agree/Strongly agree	92 (12)	-
	Neutral	8 (1)	-
	Video coaching was easier than attending in person		
	Agree/Strongly agree	92 (12)	-
	Disagree	8 (1)	-
	Number of sessions		
	About right	54 (7)	-
	Would have preferred more contact	46 (6)	-
	Duration of sessions		
	Too short	15 (2)	-
	About right	85 (11)	-
Video coaching sessions: Overall[†]	Scheduling was appropriate		
	Agree/Strongly agree	62 (8)	-
	Neutral	15 (2)	-
	Disagree	23 (3)	-
	Picture quality was acceptable		
	Agree/Strongly agree	78 (10)	-
	Neutral	8 (1)	-
	Disagree	15 (2)	-
	Sound quality was acceptable		
	Agree/Strongly agree	70 (9)	-
	Neutral	15 (2)	-
	Disagree	15 (2)	-
	Video program was easy to use		
	Agree/Strongly agree	84 (11)	-
	Neutral	8 (1)	-
	Disagree	8 (1)	-
	Technology issues delayed/prevented me from completing a session		
	Yes	46 (7)	-
	No	54 (6)	-
	Level of comfort interacting via video		

	Comfortable/Very comfortable	77 (10)	-
	Neutral	23 (3)	-
	Overall satisfaction with video coaching		
	Satisfied/Very satisfied	69 (9)	-
	Neutral	8 (1)	-
	Unsatisfied/Very unsatisfied	23 (3)	-
**Text message support**[b]	Provided me with useful information		
	Agree/Strongly agree	-	84 (11)
	Neutral	-	8 (1)
	Disagree	-	8 (1)
	Made me feel accountable		
	Agree/Strongly agree	-	54 (6)
	Neutral	-	23 (3)
	Disagree/Strongly disagree	-	23 (3)
	Increased my confidence to improve my diet		
	Agree/Strongly agree	-	22 (3)
	Neutral	-	39 (5)
	Disagree/Strongly disagree	-	39 (5)
	Increased my confidence to improve my activity level		
	Agree/Strongly agree	-	31 (4)
	Neutral	-	31 (4)
	Disagree/Strongly disagree	-	38 (5)
	Helped me achieve my weight goals		
	Agree/Strongly agree	-	8 (1)
	Neutral	-	46 (6)
	Disagree/Strongly disagree	-	46 (6)
	Helped me achieve my nutrition goals		
	Agree/Strongly agree	-	15 (2)
	Neutral	-	46 (6)
	Disagree/Strongly disagree	-	39 (5)
	Helped me achieve my exercise goals		
	Agree/Strongly agree	-	8 (1)
	Neutral	-	46 (6)
	Disagree/Strongly disagree	-	46 (6)
	Helped me eat more healthily		
	Agree/Strongly agree	-	46 (6)
	Neutral	-	31 (4)
	Disagree/Strongly disagree	-	23 (3)
	Addressed healthy eating barriers		
	Agree/Strongly agree	-	46 (6)
	Neutral	-	15 (2)
	Disagree/Strongly disagree	-	39 (5)
	Helped me be more active		
	Agree/Strongly agree	-	23 (3)
	Neutral	-	23 (3)
	Disagree/Strongly disagree	-	54 (7)
	Addressed exercise barriers		
	Agree/Strongly agree	-	39 (5)
	Neutral	-	31 (4)
	Disagree/Strongly disagree	-	31 (4)
	Useful reminder to reflect on weight goals		
	Agree/Strongly agree	-	54 (7)

	Neutral	-	23 (3)
	Disagree/Strongly disagree	-	23 (3)
	Useful reminder to reflect on exercise goals		
	Agree/Strongly agree	-	54 (7)
	Neutral	-	31 (4)
	Disagree/Strongly disagree	-	15 (2)
	Useful reminder to self-monitor		
	Agree/Strongly agree	-	46 (6)
	Neutral	-	23 (3)
	Disagree/Strongly disagree	-	31 (4)
	Personalised to my healthy eating barriers		
	Agree/Strongly agree	-	46 (6)
	Neutral	-	23 (3)
	Disagree/Strongly disagree	-	31 (4)
	Personalised to my exercise barriers		
	Agree/Strongly agree	-	39 (5)
	Neutral	-	31 (4)
	Disagree/Strongly disagree	-	31 (4)
	Number of text messages		
	Just right	-	62 (8)
	Would have preferred more	-	31 (4)
	Would have preferred less	-	8 (1)
	Overall satisfaction with text messages		
	Satisfied/Very satisfied	-	39 (5)
	Neutral	-	46 (6)
	Unsatisfied/Very unsatisfied	-	15 (2)
**Body Balance Beyond: Overall program**[b]	I would recommend the program to other women with GDM		
	Agree/Strongly agree	82 (18)	87 (20)
	Neutral	17 (4)	13 (3)
	The program met my expectations		
	Agree/Strongly agree	59 (13)	48 (11)
	Neutral	26 (6)	39 (9)
	Disagree	14 (3)	13 (3)
	I was satisfied with the program		
	Agree/Strongly agree	68 (15)	61 (14)
	Neutral	23 (5)	30 (7)
	Disagree	9 (2)	9 (2)

[a] Responses for both high and low personalisation groups combined on account of similar responses between groups (not evaluated statistically due to low group numbers).
[b] High personalisation group only
Abbreviations: BBB, Body Balance Beyond; GDM, gestational diabetes mellitus
